# Supplementary material for: Complexome profiling of the Chlamydomonas psb28 mutant reveals TEF5 as an early PSII assembly factor
Source: Plant Cell. 2025 Mar 17;37(6):koaf055. doi: 10.1093/plcell/koaf055 (PMC12164590; doi:10.1093/plcell/koaf055)
Supplement: koaf055_Supplementary_Data [file koaf055_supplementary_data.zip › psb28_interactive.zip!/plots/intensity/qualityPlots/peptides/PHT7_Cre16.g663600.t1.2.html]

### Cre16.g663600.t1.2

Trivial name: PHT7  
SeqCoverage [median]: 7.20  
Mass: 49.53 kDa  
  
 

| Sequence | MissedCleavage | Mass [Da] | wtIntensity | muIntensity | Score |
| --- | --- | --- | --- | --- | --- |
| LVPSTER | 0 | 800.44 | 1884.38 | 3655.44 | 84.24 |
| NGVPLTR | 0 | 755.43 | 0.00 | 705.46 | 70.91 |
| VLVGLGEGFAPSAATAVLAK | 0 | 1870.06 | 981.66 | 0.00 | 97.46 |
|  |  |  | 2866.0 | 4360.9 |  |
